# On the general theory of the origins of retroviruses

**DOI:** 10.1186/1742-4682-7-5

**Published:** 2010-02-16

**Authors:** Misaki Wayengera

**Affiliations:** 1 Unit of Theoretical Biology, Division of Molecular Pathology, Department of Pathology, School of Biomedical Sciences, College of Health Sciences, Makerere University, PO Box 7072, Kampala, Uganda

## Abstract

**Background:**

The order retroviridae comprises viruses based on ribonucleic acids (RNA). Some, such as HIV and HTLV, are human pathogens. Newly emerged human retroviruses have zoonotic origins. As far as has been established, both repeated infections (themselves possibly responsible for the evolution of viral mutations **(Vm) **and host adaptability **(Ha)**); along with interplay between *inhibitors *and *promoters *of cell tropism, are needed to effect retroviral cross-species transmissions. However, the exact *modus operadi *of intertwine between these factors at molecular level remains to be established. Knowledge of such intertwine could lead to a better understanding of retrovirology and possibly other infectious processes. This study was conducted to derive the mathematical equation of a general theory of the origins of retroviruses.

**Methods and results:**

On the basis of an arbitrarily non-Euclidian geometrical "thought experiment" involving the cross-species transmission of simian foamy virus (sfv) from a non-primate species *Xy *to *Homo sapiens *(*Hs*), initially excluding all social factors, the following was derived. At the port of exit from *Xy *(where the species barrier, SB, is defined by the *Index of Origin*, IO), sfv shedding is (1) enhanced by two transmitting tensors **(Tt)**, (i) virus-specific immunity (VSI) and (ii) evolutionary defenses such as APOBEC, RNA interference pathways, and (when present) expedited therapeutics (denoted e^2^D); and (2) opposed by the five accepting scalars **(At)**: (a) genomic integration hot spots, gIHS, (b) nuclear envelope transit **(**NMt) vectors, (c) virus-specific cellular biochemistry, VSCB, (d) virus-specific cellular receptor repertoire, VSCR, and (e) pH-mediated cell membrane transit, (↓_pH _CMat). Assuming **As **and **Tt **to be independent variables, **IO = Tt/As**. The same forces acting in an opposing manner determine SB at the port of sfv entry (defined here by the *Index of Entry*, **IE = As/Tt**). Overall, If sfv encounters no unforeseen effects on transit between X*y *and *Hs*, then the square root of the combined index of sfv transmissibility (√**|RTI|) **is proportional to the product IO* IE (or ~Vm* Ha* ∑Tt*∑As*Ω), where **Ω **is the retrovirological constant and ∑ is a function of the ratio Tt/As or As/Tt for sfv transmission from *Xy *to *Hs*.

**Conclusions:**

I present a mathematical formalism encapsulating the general theory of the origins of retroviruses. It summarizes the choreography for the intertwined interplay of factors influencing the probability of retroviral cross-species transmission: **Vm, Ha, Tt, As, **and **Ω**.

## Background

**Figure 1:**
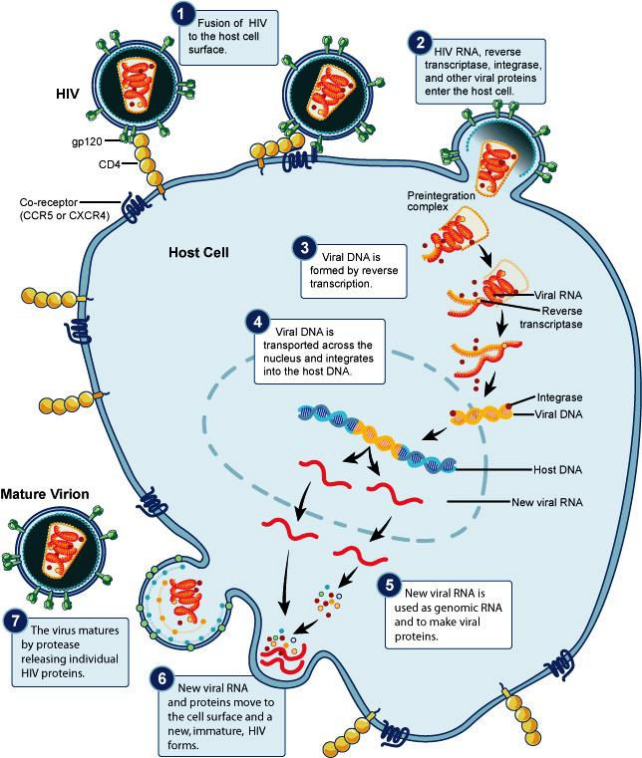
**Schematics of the retroviral replication cycle**. This figure illustrates the pathway of a retrovirus during infection of a susceptible host cell. Note the processes of (1) viral attachment to a specific receptor, (2) viral entry, (3) viral reverse transcription, (4) nuclear entry of double-stranded viral DNA, (5) viral integration into host genome, (6) viral genomic replication, (7) viral packaging and (8) budding and exit. Note that the scalars and tensors in figure 2 act at any of these steps. Source Citation [58].

The order Retroviridae constitutes a collection of non-icosahedral, enveloped viruses with two copies of a single-stranded RNA genome [1-5]. Retroviruses are known to infect avians [1] and murine [2], non-primate [3] and primate [4,5] mammals. Viruses of the order Retroviridae are unique in the sense that they can reverse-transcribe their RNA into complementary DNA, which is eventually integrated into the host genome (see Figure 1 for illustration of HIV replicative cycle) [6]. This intermediate DNA phase between RNAs may make retroviruses a valuable model for developing general virological concepts.

**Figure 2:**
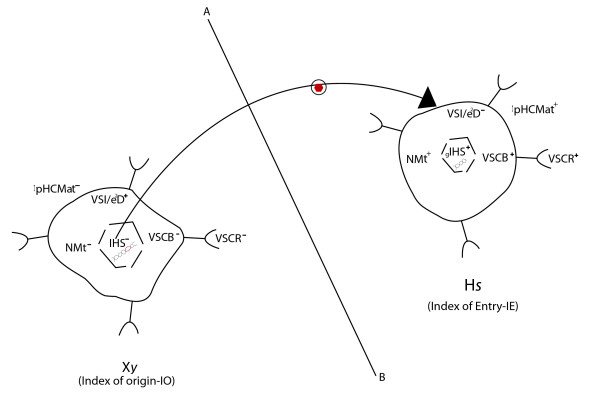
**Schematics of the imagined trajectory of a virus (sfv) jumping from one species (*Xy*) to another (*Hs*)**. The figure is based on the assumption that a retrovirus experiences: (1) an *Xy*- component of the SB denoted the Index of Origin or IO; (2) at *Hs *the index of entry or IE. The path of such a retrovirus is analogous to the trajectory of an object cast from one planet's gravitational and atmospheric field into another's. The path of such a physical phenomenon is described by Einstein's field equations of gravitation **(R_*μv*_-1/2 g_*μv*_R = 8 T_*μv*_**, where **R_*μ *_**is the Ricci Tensor, **g_*μv *_**is the metric tensor, **R **is the Ricci scalar, and T is the all important Einstein's tensor) [43]. Analogously, at the port of sfv exit from *Xy *(where SB is defined by the IO), sfv shedding is (1) enhanced by the two transmitting tensors **(Tt) **and (2) opposed by the five accepting scalars **(At)**, as described in the text. The same forces acting in an opposing manner determine SB at the port of sfv entry (defined here by the IE).

Two human retroviruses of the family Lentiviridae are known, Human Immunodeficiency Virus (HIV, which causes AIDS) [5,6] and Human T cell Leukamia Virus (HTLV a causative agent of leukemia) [4]. Emerging human retroviruses, previously undocumented in man, appear to arise by zoonotic transmission. For example, there is evidence that HIV emerged in humans after multiple independent zoonotic events involving cross-species transmissions of simian immunodeficiency viruses (SIVs) from nonhuman primates [5]. SIVs are phylogenetically very close to HIV, corroborating the role of SIV mutation (Vm) or recombination in the origin of HIV [7]. Similar cross-species transmission of retroviruses, though rarely observed among lower mammals, has been reported between felines and pumas [8,9]. These rare incidences seem to be preceded by a repeated assault (or 'attempt') on the host by the retrovirus. For example, in a recent investigation of feline immunodeficiency virus infection among bobcats and pumas in Southern California, Franklin et *al*. [8] provide evidence that cross-species infections have occurred frequently among these animals leading to the eventual transmission of the virus (FIV) to puma. The above data imply the existence of a biological restriction on cross-species retroviral transmissions, the species barrier (SB) [8]. For the purposes of this work, SB was defined as a biological barricade that inherently restricts cross-species transmission of retroviruses but, when jumped, enables such transmission. The repeated host assaults needed by the retrovirus to achieve cross-species transmission may also suggest that a level of host adaptation (as well as retroviral mutation or recombination) is required to effect the SB jump. This is consistent with the postulates of an earlier hypothesis I advanced to explain origins of retroviruses [10,11].

It is well established that repeated contact between a potential new and a known reservoir host plays a role in breaching the SB, but the dynamics of the underlying molecular mechanisms remain ill-defined. Current understanding may suggest that a threshold of retroviral load is needed to achieve inoculation, or viral mutation (Vm) and possibly new host adaptation (Hm) is needed to achieve retroviral cross-species transmission [8-12]. All in all, recent evidence for the regular transmission of primate retroviruses suggests that zoonosis, *per se*, may not be the rate-limiting step in pandemic retrovirus emergence, and that other factors such as viral adaptation are probably important for successful cross-species transmission and a human pandemic [12]. Vandewoude et *al*. [9] used an experimental model to establish that although domestic cats (*Felis catus*) are susceptible to FIVs originating from pumas or lions, the circulating virus is reduced to nearly undetectable levels in most animals within a relatively short time. This diminution of viral load was found to be proportional to the initial viral peak, suggesting that the non-adapted host successfully inhibits normal viral replication, leading to replication-incompetent viral progeny. The possible mechanisms proposed for such restriction of cross-species infection in natural settings include: (1) lack of conducive contact between infected and shedding animals of different species; (2) lack of a suitable receptor repertoire to allow viral entry into susceptible cells of the new species; (3) a sufficient difference in cellular machinery between the new and the primary host to preclude viral replication; (4) intracellular restriction mechanisms in the new host that limit viral replication; (5) ability of the new host to raise sterilizing adaptive immunity, resulting in aborted infection and inability to spread infection among con-specifics; or (6) production of defective or non-infectious viral progeny that lack the cellular cofactors required to infect conspecifics [5]. Overall, these data support the view that there is a unique requirement for retroviral fitness (Vm) and for host adaptability (Ha) to effect the SB jump. The same work also points to the existence of intracellular restriction mechanisms for cross-species retrovirus transmission (hereafter denoted *transmitting tensors*, **Tt**) as well as intracellular mechanisms that can promote inter-species transmission of retroviruses (hereafter denoted *accepting scalars*, As) [8,9,12].

The purpose of this work was to derive a mathematical formalism that integrates and expresses the molecular interplay among Vm, Ha, Tt and As during enhancement or breach of the SB when retroviruses are transmitted across species. On the basis of an arbitrarily non-Euclidian geometrical "thought experiment" involving the cross-species transmission of simian foamy virus (sfv) from a non-primate species *Xy *to *Homo sapiens *(*Hs*), initially excluding all social factors, the following was derived. At the port of sfv exit from X*y *(where SB is defined by the *Index of Origin*-IO); sfv shedding is (1) enhanced by the two tensors **(Tt): **(i) virus specific immunity (VSI) [13-15] and (ii) evolutionary defenses such as APOBEC [16-19], Tripartite Motif (TRIM) family [20], interferon-induced transmembrane protein BST-2 (CD317; tetherin) [21], RNA interference pathways [22-24], plus, where present, expedited therapeutics (all denoted e^2^D); *and *(2) opposed by the five Accepting scalars **(As): **(a) genomic integration hot spots-gIHS [25-33], (b) nuclear membrane transit **(**NMt) vectors[6], (c) virus specific cellular biochemistry-VSCB[6], (d) virus specific cellular receptor repertoire-VSCR [34-39], and (e) pH mediated cell membrane transit-(↓_pH _CMat) [40-42]. The scalar function, as used here in biological space-time, differs from its physical analogue in that it exhibits both magnitude and direction (in contrast to physics, where scalars only have magnitude) that are equal and opposite to the tensor function. Assuming **As **and **Tt **to be independent variables, **IO = Tt/As**. The same forces acting in an opposing manner determine SB at the port of sfv entry (defined here by the *Index of Entry*, **IE = As/Tt**). Overall, if sfv encounters no unforeseen effects on transit between *Xy *and *Hs*, the square root of the combined index of sfv transmissibility (√**|RTI|) **is proportional to the product IO* IE (or ~Vm* Ha* ∑Tt*∑As*Ω); where Ω is the retrovirological constant, and ∑ is a function of the ratios Tt/As or As/Tt for this particular arbitrary event of sfv transmission from X*y *to H*s*.

## Methods and approach

### The "thought experiment"

**First, **to contemplate the mathematical scope of the dynamics of retroviral cross-species transmission, I concocted a thought experiment involving the transmission of a retrovirus-simian foamy virus (sfv) from the arbitrary non-human primate species *Xy *to *Homo sapiens*. The system was imagined to exclude all social factors such as contact and contact repetition; it was assumed that only biological factors influence retroviral cross-species transmissions, until another constant is introduced that may also integrate social factors, the *retrovirological constant*. In this "thought experiment", sfv must first break free from the influence of the net of molecular determinants of SB in *Xy *(the component of SB here being derived as the *Index of Origin*, **IO**) before entering *Hs *by similarly overcoming the SB determinants there (the relevant component of SB being defined by the *Index of Entry*, **IE**).

In order to derive the pathway of sfv mathematically, I observed that only the kind of non-Euclidian geometry that represents curvature in space-time may suffice. This led me to recruit an unlikely-seeming comparison between physical and biological phenomena (unlikely since the former are mostly concerned with constants while the latter largely involve dynamic processes that differ among species and individuals). Specifically, I re-envisaged the dynamics of sfv cross-species transmission as analogous to those of a comet traveling from Mars to earth. Such a comet must first break through the gravitational and atmospheric fields of Mars (analogous to the point when sfv breaks free of the net effect of IO operating in *Xy*) and then move through free space until it breaks through the earth's atmospheric and gravitational fields (analogous to the point at which sfv breaks through the IE in *Hs*) (see figure 2). The path of such a comet is best described by Einstein's field equation of gravitation **(R_*μv*_-1/2 g_*μv*_R = 8 T_*μv*_**, where **R_*μ *_**is the Ricci Tensor, **g**_*μv *_is the metric tensor, **R **is the Ricci scalar, and T is the all-important Einstein's tensor) [43-45]. The dynamics of retroviral cross-species transmissions do not really resemble such physical phenomena, but this arbitrary comparison crucially led to the insight that non-Euclidian tensors may similarly be used to represent the SB variables **Vm, Ha, Tt **and **As **at the ports of both sfv origin and exit [46].

Tensors are vectors that contain multiple independent variables possessing both direction and magnitude. In Euclidian geometry, increases in the number of components account for various dimensions of visualization. For instance, in 2-D, every tensor has three components; six components are integral in a 3-D tensor, and 10 in a tensor of 4-D (the realm of physical space-time) [46]. The non-Euclidian space-time tensors that Einstein used to derive his field equations of gravitation have over 16 independent components [43-45]. Thus, to assume that cross-species retroviral transmission assumes a path closely similar to that of the physical phenomena has implications for the nature of the variables **Vm, Ha, Tt **and **As**: (i) **Vm, Ha, Tt **and **As **are non-Euclidian tensors in 4-D comprising 16 or more components; (2) they are covariant in nature, meaning that there can only be one possible finite value for each. The influence on SB jump dynamics of a change in the finite value of any of the 16-plus components is balanced by reciprocal changes in the others, ensuring the constancy of **Vm, Ha, Tt **and **As**. The unique advantage of this approach is that only a few of the components need to be known for a mathematical formalism of the theory of retroviral transmission to be obtained. This is important because not all the molecular determinants of retrovirus species cross-species transmission are known.

### Annotation of the non-Euclidian biological tensors/scalars Vm, Ha, Tt and As

**Second, **to annotate the components of the non-Euclidian tensors and scalars operating in this imagined scenario of sfv cross-species transmission (the full composition may remain uncertain because many determinants are still poorly understood), I followed sfv on its imagined path through each compartment of *Xy *and *Hs*, defining and positioning the currently-known biological determinants of the transmission process (see Figure 2). At the port of sfv exit from *Xy *(defined by IO), sfv shedding is (1) enhanced by the two transmitting tensors **(Tt) **and (2) opposed by the five accepting scalars **(At) **explained above. Continuing the thought experiment, the same factors are bound to operate at the port of sfv entry into *Hs *(synonymous with IE), except that what were annotated as transmitting tensors become accepting scalars, and vice versa. Because each individual tensor and scalar was annotated to be largely compartmentalized, it seemed appropriate to consider rules of multiplication or fractionation to govern their future combinations, since mathematically they may be considered mutually independent. Hence, assuming **As **and **Tt **within the same host to be independent variables, then **IO = Tt/As**. (When similar forces act in an opposing manner to determine SB at the port of sfv entry, IE = **As/Tt**).

Overall, two major assumptions were made throughout these derivations. *First*, only biological factors were considered, leaving social factors such as contact and contact repetition aside; several existing models deal with those [47-53], and a subsequently introduced covariant, the retrovirological constant, may be used to account for them. *Second*, I assumed that the retrovirus sfv experiences no uncertain influences of any mode or origin between its ports of exit and entry [46]. This is obviously a major presumption, especially since most effective "public health control measures" would best be situated between those ports.

**Figure 3:**
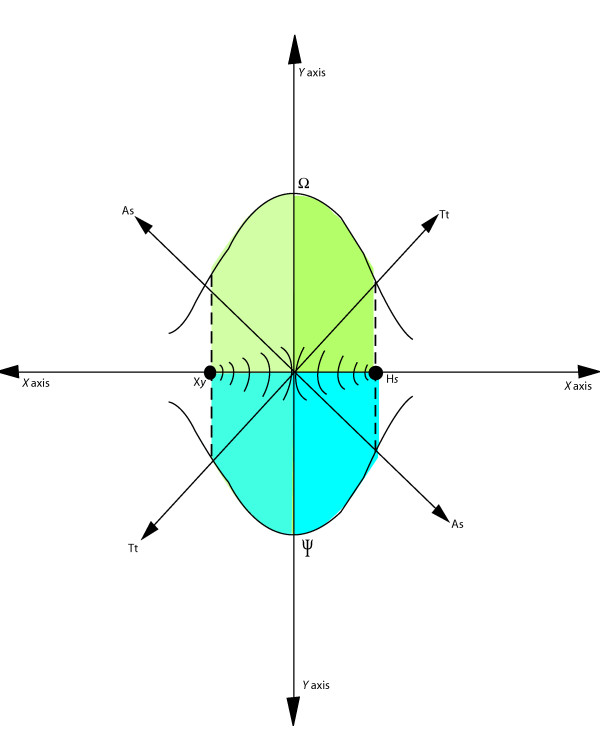
**Schematics of the geometrical relationship between the retrovirological constant Ω and the retrovirological window ψ**. This figure illustrates the variation of retrovirological constant in both its positive and negative realms (the retrovirological window **ψ **is geometrically supposed as the mirror image of the retrovirological constant Ω around *Xy *and *Hs*, or x axis). Classically, the boundaries possibly define the highest point of quorum sensing and signal transduction between retroviral events in and around the space between *Xy *and *Hs*. Within the same is represented: (1) retrovirological fields (which are predicted to intertwine most when the space between *Xy *and *Hs *approximates 0), (2) Variation of the transmitting tensors **(Tt) **and accepting scalars **(As) **around **ψ/Ω **and **X*y*/H*s***. This is Wayengera's advanced graph of the physico-mathematics of retrovirology. The x-axis represents space and the y-axis time. The graph itself, though 2-D, is a one-dimensional visualization of space-time within the retrovirological field(s). The parabolic nature of Ω [(y - k)^2 ^= 4a(x - h)], apparent in this graph, results from a sort of reversal in time when sfv ceases to break free of *Xy *and embarks on *Hs *entry. All points on and within **Ω **and **ψ **may be denoted as the path of least action (when retroviral transmissibility is most likely and predictable, i.e. when retroviral fields are intimately intertwined). Also inherent in the graph is the 'wave-particle duality' of retrovirology and biological phenomena as a whole. The areas under **Ω **or **ψ **are to be denoted probability densities or orbitals for RTI, Va, Ha, ∑Tt and ∑As.

What are arbitrarily annotated as tensors and scalars represent, in real biology, innate or acquired ecological responses of the retrovirus/host to variations in population-wide dynamics, and some may be subject to adaptation. The resulting unpredictable behavior of biological systems, in contrast to physical phenomena, underlines the fact that possibly no single physico-mathematical system can portray events in biology sustainably over time, unless it (a) leaves open a window to allow for uncertainty arising from biological unpredictability, and (b) recognizes retroviral transmission as analogous to a dual wave-particle phenomenon. This view led to the concept of a retrovirological window (discussed below) and use of a mosaic of quantum and relativistic approaches [54] to define qualitatively the range of space-time in the retrovirological fields over which the equations advanced may be accurate (see Figure 3).

### Derivation of the equivalent of IO and/or IE

From the arbitrary annotation of forces influencing sfv *Xy *exit or *Hs *entry, it may be stated mathematically that:(i)

However, **As **may currently be represented as proportional to:(iii)

And **Tt **may mathematically be denoted as follows:(iv)

From equations iii and iv, equations i and ii become re-expressible as v and vi below, respectively. Two further major assumptions are made here to remove the proportionality sign and replace it with an equal sign.

• **In the first instance, **it was necessary to introduce within the transmitting tensors an arbitrary constant of innate or acquired viral fitness specific to the retrovirus concerned, denoted **λ**. At the ports of exit and entry, retroviral fitness is denoted respectively **λ^0 ^and λ'**. These factors serve to illustrate that, even if viral mutation (or phenotypic adaptability) is already noted as a major player in retroviral cross-species transmissibility, it is tailored to the retrovirus in question; some retroviruses are predictably more mutable than others. Also, because the several non-Euclidean components of each transmitting tensor (compartments VSI and e^2^D used here) remain ill-defined, arbitrary multiplying factors were introduced for each transmitting tensor compartment, π1 and π2 for VSI and e^2^D respectively; their integral products are **π^0 ^or π' **within the host of origin and that of entry respectively.

• **On the other hand**, for the accepting scalars, a constant for specific host adaptability (φ) was necessary to formalize the dynamics of retroviral cross species transmissibility correctly and comprehensively; **φ^0 ^**and **φ' **for *Xy *and *Hs *respectively. In addition, as for the tensors, the relationships among the five independent accepting scalar compartments listed should have a governing proportionate factor for each (since their full composition is apparently unknown): κ1, κ2, κ3, κ4 and κ5 respectively for **VSCR, ↓_pH_CMavt, VSCB, NMt **and **gIHS**; the derivative products are **κ^0 ^**and **κ'**.

Hence,(va)

may be approximated to:(vb)

Similarly,(via)

may be approximated to:(vib)

Formulation of the integral equation: the relative transmissibility index (RTI)

From equations v.b and vi.b, the relative transmissibility index (RTI) may be mathematically formalized as:(vii)

Substituting from equations v.b and vi.b:(viia)

Further major simplifications may now be introduced:-

• First, **λ'/λ^0 ^**may be considered equivalent to specific viral mutability: **Vm**

• Second,**φ^0^/φ' **is the inverse of host mutability, termed host adaptability: **Ha**

• Third, the complex factor π^0 ^[VSI*e^2^D]^0^*1/(π' [VSI*e^2^**D]') **equals the effective Net Transmitting tensor: **∑Tt**

• Forth, the complex factor κ'(VSCR*↓_pH_CMat*VSCB*NMt*GIHS)'*1/[κ^0^(VSCR*↓_pH_CMat*VSCB*NMt*gIHS)^0^] represents the effective net accepting scalar:**∑As**

As used here, **∑ **denotes a function of the ratio Tt/As for sfv transmission from *Xy *to *Hs*, and not its usual formal mathematical implication of summing.

Hence,(viii)

In order to replace the proportionality sign with an equal sign, a new constant, the retrovirological constant **(Ω)**, is introduced. This brings us to the final equation advanced for the general theory of retrovirology:(ix)

Observe that, if one alternatively purposed to consider **Tt **and **As **operating within the same host as dependent variables (a scenario I disregarded since it makes the biological phenomenon nearly homologous to physical phenomena), then, by maintaining Vm and Ha as independent, the same equation ix may be re-phrased as: **|RTI|**= **∑**(Tt-As)_Xy _*∑(As-Tt)_Hs _* Vm*Ha*Ω; in which case ∑ retains its mathematical meaning of summing.

Is this just another mathematical attempt at biology, or it is something that may add to our knowledge of retrovirology and possibly other infectious pathogen transmission dynamics? It is an enormous and serious challenge to simplify and unify retrovirology. I discuss below the ramifications I have so far seen of the proposed formalism; readers may find other insights. In addition, I suggest experiments that may be undertaken to test how well this equation represents retroviral cross-species transmission dynamics.

Additional modifications are made to the formalism as I re-visualize it in the light of the existing literature in physics, mathematics and retrovirology.

## Discussion

The mathematical formalism of the theory of the origins of retroviruses presented above suggests that retroviral cross-species transmission results from a random yet geometrically predictable intertwining of Vm, Ha, Tt, As, and Ω, a pattern consistent with the four postulates of the evolutionary adaptation cross-species (EACS) hypothesis I previously advanced to explain the origin of human viruses, the scope of which I have since limited to retroviruses.

*First *(P_1_), emerging and re-emerging retroviruses exist before they are isolated or there is evidence that they cause human disease. They existed in previous hosts called "reservoirs", mostly wild game species, on which they depended for the virus-host cell interaction necessary for survival - making all retroviruses zoonotic in origin.

*Second *(P_2_), with an increased change in variables among the reservoirs and chance of contact with a new host (humans), these retroviruses adapted, possibly but not necessarily through mutation, recombination and re-assortment to yield new strains with better fitness to use human cells for replication.

*Third *(P_3_), for all newly emerging retroviruses, the most susceptible new hosts are those whose cellular biochemistry and genetics favors establishment of the virus by coding for and producing the necessary energy, metabolites and most (or in some cases all) the enzymes required for replication of the adapted new strain. Depending on the endogenous tissue specificity (fitness) exhibited by a retrovirus; however, retroviral cross-species jumps are possible between host species of variable biochemical and genetic homology.

*Fourth *(P_4_), various mechanisms of interaction between the previous and new (human) hosts are required to effect cross-species retroviral transmission. The mechanisms of transfer may be **either **direct (e.g. via human consumption of NHP game meat for simian immunodeficiency viruses such as SFV, or transplantation with porcine tissue for PERV-xenosis) **or **indirect, by vector transfer (a predicted scenario that may occur, say, with retrovirus-based bio-weaponry, discussed below) [10,11]. The general equation derived above suggests potentially interesting though not yet fully comprehensive ideas on: (1) the possible ramifications of this physico-mathematical formalism of retrovirology and (2) the experiments that may be needed to test it.

## Ramifications of the equation of the theory of retrovirology

### Insights into the overall dynamics of cross-species transmission

From the final equation of retrovirus origin, the imaginary scenario involving transmission of the simian foamy virus (SFV) from the non-human primate species *Xy *to *Hs *may be considered as follows: whenever the net biological Tt and As within the animal host *Xy *is greater (in favor of Tt within *Xy*) than the corresponding value in *Hs*, there is a greater probability of cross-species transmission (best visualized using the variant of the equation that assumes Tt and As operating within the same host to be dependent variables, **|RTI|**= **∑(**Tt-As)_Xy _*∑(As-Tt)_Hs _* Vm*Ha* **Ω**). Conversely, greater net Tt and As in *Hs *than in *Xy *(in favor of Tt within *Hs*) will disfavor sfv transmission. Thus, intense retrovirus (sfv)-specific immune responses in the animal host will enhance retroviral shedding and hence cross-species transmission, while similar responses in *Hs *will restrict viral tropism there. In general, any change among the covariants **Vm, Ha, Tt, As **and **Ω **that makes the **|RTI| **> 1 will favor the specified direction of cross-over of the arbitrary retrovirus sfv (from *Xy *to *Hs*), while co-variations making **|RTI| **< 1 will disfavor it.

Wherever there is VSI (annotated as Tt), the natural reservoir elicits no retrovirus-specific immune responses (and VSI is possibly always zero or one). However, within the same natural reservoir setting, where retroviruses live harmoniously with the host, the equation predicts that artificial stimulation of virus-specific immune responses will favor viral shedding. Whether interactions between X*y *and H*s *can elicit VSI within X*y *remains to be established, but this would make retroviral shedding an adaptive response mounted by X*y *to protect its niche from encroachment by H*s*, and the retroviruses themselves would be commensals with guardian characteristics. This implies that vaccination of the reservoir host, unless it entirely eliminates the retrovirus, cannot reduce the risk of retroviral cross-species transmission and may indeed enhance it. Because the natural reservoir elicits no immune responses to the pathogen, this may explain the difficulties and discordance of results obtained by filovirus-specific IgG/M antibody detection tests and virus capture assays during the search for a natural reservoir of the re-emerging filoviruses Ebola and Marburg. Applying the field equations of retrovirology, it can be predicted that no virus will be detected within the natural reservoir, even when the filovirus is present, because there are no filovirus-specific immune responses. This renders assays of pathogen immune responses within the host inappropriate for studies that aim to identify the natural hosts of any pathogen (and techniques for the isolation of the pathogen Koch's style must continue to be considered). The equation of retrovirology also suggests that the 'purpose' of repeated zoonotic transmission of a retrovirus, such as that involving various SIV isolates reported between non-human primates and humans, is as follows. (a) It fine-tunes the new host's adaptability to sustain actively facilitated retroviral replication including (i) the selective adaptation of a permissive receptor repertoire where absent, (ii) recruitment of alternative biochemical pathways, and (iii) development of mechanisms for inhibition or outright evasion of any inherent inhibitory mechanisms within the target host such as APOBEC [16-19], interferon-induced transmembrane protein BST-2 (CD317; tetherin) [21], TRIM [20] and RNAi [22-24], present within most mammals. (b) It facilitates retroviral mutation or recombination. These two teleological reasons for repeated retroviral infections of a new host before the ultimate jump of the SB allow for the evolution of (1) host adaptations and (2) viral mutations or recombinations that will interact to make the host and virus fitted to cohabit [12,47].

This implies that a "bio-weapon" may be developed in the laboratory by continuous cycles in which an acutely fatal retrovirus of zoonotic origin is co-cultured in human cell lines, rendering the human cells permissive to that retrovirus tropism. The same procedure may be used to select an appropriate animal carrier or "vector", say chickens, pigs, or even cows. Herein, shedding of the retroviral bio-agent may be enhanced by vaccination of these vector-hosts if they are appropriately adapted to act as natural hosts. Several other models for retrovirus-based bio-weaponry are possible, including starting with a known human retrovirus such as HIV and recombinantly engineering it to be acutely fatal (say by pseudo-enveloping it with or enabling it to express Ebola/Marburg gp1, 2, the major pathogenic protein of filovirus hemorrhagic fever). Additional modifications such as altering the transmission dynamics of the retrovirus from contact with infected body fluid to air- or water-borne transmission would make it more damaging, though it is not immediately clear how that could be achieved.

More peacefully and productively, the mathematical formalism of retrovirology advanced here also underscores strategies for avoiding or mitigating the impact of retrovirus-based bio-weapons, such as the development of therapeutic interventions and avoidance of contact (see below).

### Retrovirological fields and their action

From the final equation of the general theory and the platform of "thought experimentation" that relates biological and physical phenomena, 'retrovirological fields' around hosts may be imagined, analogous to gravitational and atmospheric fields around planets. This proposal arises from, and in support of, the assumption that a retrovirus crossing from one host to another encounters two barriers. Although the covariant nature of the tensors **Vm, Ha, Tt **and **As **makes their overall finite value appropriately covariant for deriving retrovirological fields mathematically, the real wave (or possibly quantum) pattern of such fields is best accounted for in the parabolically covariant nature of the retrovirological constant, as discussed below. In brief, the scenario is as follows: just as the mathematical formalism of retrovirology advanced here predicts that retroviruses within wild game (constantly adapting) are ever mutating (to expand their fields of retrovirological operation), so it is predictable that retrovirological fields will never be constant. This invites the question: "how may retroviruses themselves influence retrovirological fields, or vice versa?" From the equation of retrovirology, the central dogma of retrovirus transmissibility is: "Retroviral mutations serve to induce changes (by either approximation or distancing) within the retrovirological fields operating around hosts in a similar way to host adaptability". Hence, just as in the relationship between matter and space-time - "matter tells space-time how to curve, and curvature in space-time tells matter how to move" [43-45] - I believe that 'retroviruses and their hosts tell retrovirological fields how to change, and changes in retrovirological fields tell retroviruses and hosts how to mutate, adapt or transmit' [47].

Evidence in support of this interplay arises from data that link human activities such as hunting, mining, etc., which bring man into close contact with wild game harboring retroviruses such as HTLV-3/4, with human infection by the same [3,7]. In other words: the general theory of retrovirology provides the choreography for an intertwined dance of chance of retroviral cross-species transmission, **Vm, Ha, Tt, As**, and **Ω **(see Figure 3 for illustration).

### The retrovirological constant and its parabolically covariant nature: highest peak at the closest intertwining of retrovirological fields

Perhaps the greatest theoretical predicative power of the mathematical formalism of origins of retroviruses lies in its ability to elucidate the nature of a still-ambiguous constant, the retrovirological constant. Although the mathematical significance of the retrovirological constant in ensuring a balance between both sides of the equation is apparent, its finite value is not. Nevertheless, several predictions can be made about its nature and scope.

*First*, if the retrovirological constant is a non-Euclidean tensor as stated in equation **ix**, then, assuming that biological space-time is 4-D, it comprises 10 or more independent components [43-46]. Therefore, because the equation of retrovirology was derived purely on the basis of biological determinants of retroviral cross-species transmissions, the retrovirological constant may also be taken to incorporate several social determinants of retroviral transmissibility including mode of contact, contact repetitions [48-52] and the basic reproductive number of the retrovirus (R_0_) [53]. In other words, the retrovirological constant is itself a non-Euclidian tensor that allows space for as many currently unknown factors in retrovirology as may be conceived. Alternatively, because the equation advanced says nothing about the virulence of sfv once it successfully integrates into the cells of the new host, *Hs*, a virulence factor may appropriately be integrated into the constant.

*Second*, just as the tensors and scalars **Vm, Ha, Tt **and **As **are predictably covariant, the retrovirological constant **Ω **is similarly covariant. The covariance of **Ω **implies that, as some of its components change, there is a tactically balanced adjustment in others so that at any given time its tensor value remains constant [43-46].

*Third*, since the probability of retroviral transmission increases as *Xy *and *Hs *become more closely intertwined, we have assigned a "parabolic" pattern to the influence of **Ω **on the overall cross-species transmission dynamics of retroviruses. This implies that **Ω **is highest when *Xy *and *Hs *are closest, xeno-transplantations and culinary habits being better promoters of retroviral transmissibility than mere side by side contact. *In situations of first retrovirus attempt on Hs, it should be noted that only Xy will have a field, the field around Hs being absent *(, unlike the image is presented in Figure 3). Concealed but obviously inherent in the parabolic nature of Ω is the 'dual wave-particle' pattern of the imaginary retrovirological fields discussed above (see Figure 3 for a detailed illustration). In the light of further discussions (below) about the interactions between the retrovirological constant and the "retrovirological window", the retrovirological constant can itself be observed to demarcate the only true realms in which retrovirus cross-species transmission dynamics are predictably influenced by physical transmission.

### On the need for and nature of the retrovirological window (ψ)

As observed in the Methods and Approach section (*Annotation of the non-Euclidian biological tensors/scalars*), what were arbitrarily annotated as physical tensors and scalars do not represent biological phenomena realistically, since such phenomena are simply innate or acquired ecological responses of the retrovirus/host to variations in population wide dynamics, and are probably subject to adaptive alterations. This led me to define the true realms of space-time within retrovirological fields in which the equation of retrovirology can be said to be most predictive of retrovirus cross-species transmission dynamics. To do so, I introduced the concept of a retrovirological window (**ψ**). If the retrovirological constant **Ω**, as suggested above, may be defined graphically as the boundary of positivity in which the equation of retrovirology best predicts real events of retrovirus cross-species transmission dynamics that are most closely analogous to physical (non-biological) phenomena, then the retrovirological window **ψ **is the mirror image of **Ω **(see Figure 3 for a geometrical illustration of relationship between **Ω **and **ψ)**. If **ψ **is simply a mirror image completing the 2-D representation of what are actually 4-D psi-formalisms of Ω, it is possibly not required in the equation. However, to account for its invisible influence on the overall dynamics of retroviral cross-species transmission, **ψ **may be integrated by rephrasing the equation to **^ψ^√|RTI| **= Vm *Ha*∑Tt*∑As*Ω or **|RTI| **= (Vm*Ha*∑Tt*∑As*Ω)**^ψ ^**so that, in 2-D visualization (where **ψ **= 2), each tensor or scalar Vm, Ha, ∑Tt, ∑As and Ω always has both a positive and negative true value.

This approach is borrowed from quantum mechanics, where the linear representation of the path of an electron or photon is represented mathematically by squaring the amplitudes to yield pulses in 2-D (see Figure 3) [54]. This also implies that, on a scale of X*y *to H*s*, the integral of each of the normalized tensor and scalar functions Vm, Ha, **∑**Tt, **∑**As and Ω is always one. Using the alternate approach that assumes dependency of Tt and AS within the same host, **|RTI|**= {**∑(**Tt-As)_Xy _*∑(As-Tt)_Hs _* Vm*Ha*Ω}^2^.

### On the nature of space-time within biological systems

Despite what may seem an apparent success in using non-Euclidian geometry to derive equations of retrovirology, several questions remain unanswered. *First*, if space-time in physics is four dimensional [43-46], is it appropriate to hold the same for biological phenomena, or are adjustments needed? The significance of this question is that, in Euclidean geometry, each tensor or scalar for (a) 2-D space-time has three components, (b) 3-D has six components, and (c) 4-D has 10 independent components [46]. In the non-Euclidean geometry that Einstein adopted for space-time when deriving his field equations of gravitation (general relativity), each tensor had 16 components [43-45]. The question therefore becomes rational because, given that non-Euclidean geometry was borrowed to arrive at a general theory for the origins of retroviruses/retrovirology (meaning, we assumed biological space-time to be 4-D), the possible number of space-time dimensions in biology and the number of its components are open to inquiry.

*Second*, regardless of its finite composition, are the determinants of space-time in retrovirology limited to **Vm, Ha, Tt, As**, and **Ω **or there more?

*Third*, are events in retrovirological space-time best regarded as particles, waves or dual? As shown in Figure 3, I have been led to adopt a 'dual wave-particle' representation of retroviral cross-species transmission dynamics [54].

These and possibly other issues that remain unclear leave the close-to-real physico-mathematical representation of biology a matter for further inquiry.

## Hints on testing the equation of the theory of retrovirology

Several unabridged gaps in experimental retrovirology are predicted by this unifying "equation of retrovirology", but many may be elucidated experimentally, underlining the need for further experimentation on the pathway of retroviral cross-species transmission to make the equation practically useful.

(1) Although data on the requirement for zoonotic viral mutations to achieve infection of humans are scanty, further experimental evidence is necessary to affirm the influence of Vm and Ha on the overall dynamics of retroviral cross-species transmission.

(2) The scope of both accepting scalars and transmissive tensors remains rather ambiguous and must be clarified. In addition, the correlation of changes in the individual components of the scalar and tensors with retroviral transmission dynamics must be corroborated by in-situ or in-vivo experimentation.

(3) Innovative techniques for the experimental quantification of components of both scalars and tensors affecting retroviral cross-species transmission are needed to define the finite measure of the retrovirological constant (**Ω**), even before we contemplate what its components are or may be. Perhaps field retrovirology may benefit from the following insights and propositions.

### (a) Experimental evidence for the requirement of Vm and Ha in retroviral cross-species transmission

Completion of sequencing of several organismal genomes along with technological advances such as computation, software and web-based repositories of omes make it possible to obtain data not just on various mammalian and retroviral species genomes, but on their proteomes, transcriptomes, metabolomes etc. [55]. Although they are not yet appropriately unified to support retroviral work, such repositories, based on the entire omes of the virus and hosts before and after the establishment of competent retroviral cross-species transmission, can enable in-silico comparisons of viral and host omes to be made on either side. For instance, the retrovirus resource at NCBI's resource center for retroviruses (available at http://www.ncbi.nlm.nih.gov/retroviruses/) forms a useful starting point for constructing such virtual databases. Data obtained in these sorts of bioinformatics experiments will inform whether retroviral mutations such as those seen with pandemic influenza of swine or avian origin are always required to achieve retroviral transmission. In the same way it will enlighten us of the need for host adaptability at the molecular level (e.g. immunological), no matter how small.

### (b) Alternative *in-silico, in-vitro*, or *in-situ *experiments to derive evidence in support of the role of variations within individual components of the accepting scalars and transmitting tensors in reservoir and new host

Affirmation of the relationship between the frequency of genomic integration hot spots (gIHS) and the rate of retroviral genome integration may be aided by using 3-D-based bioinformatics searches of 3-D host genomes or transcriptomes other than primary structure-based analysis[56,57]. Data from several mammalian genomes support the possible conservation of some candidate retrovirus integration hot spots such as LINE elements, Alu, CGp transcriptional sites and topoisomerase cleavage sites. However, in the light of uncertainty in existing data [25-33], it is necessary to determine experimentally whether retroviral gIHS are similar for all retroviruses in all hosts or whether they differ from one retrovirus or host to another, as current evidence suggests. Real time expression profiles of various virus-specific immunity (either by targeted Ellispot or proteome-wide association studies, PWAS) and evolutionary defenses such as RNAi (say, by transcriptome-wide association studies, TWAS), when correlated with the probability of viral integration and appropriately controlled for, may elucidate both the direction and the magnitude of their effect on retroviral cross-species transmission. **Vm **may be measured as the ratio **λ'/λ^0 ^**and **Hm **as the ratio **φ**^0^**/φ'**, finite values of **λ **and **φ **being measured by automated sequencing and denoted as the number of unnatural base variations in the retrovirus and the host genome size in nucleotides.

## Conclusions

Once such suggested and appropriately standardized experiments and techniques for the quantitative and qualitative determination of all SB tensors and scalars have been conducted, then, using real time data obtained from sampling of molecular epidemiology cohorts such as those recently described by Vandewoude *et al*. [9], one may not only test the theory advanced, but derive the finite equivalents of the retrovirological constant (**Ω**).

The practical value of the mathematical formalism proposed in this paper can then be assessed. This should include, I suggest, an inquiry into whether (1) the same model of inter-species transmission of infectious agents (zoonotic origins) may be extended without modification to the inter-species dynamics of other infectious agents, and (2) intra-species transmission dynamics of all human infectious agents can be predicted by a modified version of that model.

## Competing interests

The authors declare that he has no competing interests.

## Authors' contributions

WM conceived the hypothesis behind this work, designed and undertook the synthesis and derived the deductions. WM also wrote the final draft of the manuscript.
